# PhysPrac: a custom physiotherapy application

**DOI:** 10.1093/jhps/hnv015

**Published:** 2015-02-25

**Authors:** Austin V. Stone, Allston J. Stubbs

**Affiliations:** Wake Forest School of Medicine, Orthopaedic Surgery, Medical Center Boulevard, Winston-Salem, NC 27157, USA

The timely creation of a patient-specific physical therapy and rehabilitation program is a challenge. PhysPrac by Active Health Tech (2014, USD$ 7.99 on the AppStore) provides an inexpensive point-of-care solution for rapidly generating a custom rehabilitation protocol. The mobile platform allows users to select from an existing evidence-based exercise database or use custom exercises to build a patient-specific rehabilitation program. A color PDF protocol can be directed to the patient from within the application. The ability to create a personalized rehabilitation program meets the needs of the hip preservation surgeon for pathology-specific rehabilitation and improved therapy communication.

The therapy database is extensive and includes over 120 exercises that can be grouped by region or by goal, with approximately 40 directly related to the hip and thigh and additional ones focusing on core strengthening and proprioception. Each selected exercise includes simple pictorial illustrations along with a detailed description for the patient. A ‘Key Points’ commentary identifies common exercise pitfalls to help the patient maximize its effectiveness. The number of sets, repetitions and resistance is standardized but is quickly customizable with a few screen taps. Each exercise picture may be modified with illustrations imported from your device, which offers improved utility for hip-specific rehabilitation. Your practice logo and information may be added to the PDF sent to the patient.

A distinct advantage of the application is the ability to save protocols and utilize for future patients. This feature saves time for commonly used surgeon-specific programs but exercises can be easily added or modified to generate the optimal plan for individual rehabilitation goals. Patient-specific optimization of rehabilitation plans, such as targeting hip flexor or abductor weaknesses, may help address the complex pathology frequently encountered by hip preservation surgeons. On return visits, a new therapy program could be sent based on the patient’s progression. This strategy reduces the amount of variability when the patient is working with a physiotherapist who is not familiar with a surgeon’s protocols.

Included with purchase of the application are all of the aforementioned abilities and 50 patient emails. Additional emails may be purchased for a nominal fee in small blocks or unlimited emails for USD $39.99. As the application does not store patient medical information, it should be compatible with privacy laws.

The application could be improved with an associated client application that reminds patients to perform exercises and track progress. PhysPrac is currently limited to Apple devices; however, the publisher, Active Health Tech, is launching a new product in the future as a cloud-based system. This updated application will offer greater services for patients and may be more useful for higher volume practices. The company is offering free migration from the existing application to the new cloud-based service once available.

Ultimately, this physiotherapy application offers high value through its ability to quickly and easily generate custom programs tailored to the hip surgeon’s preferences. Users of this new technology will benefit from future software upgrades and, ideally, improved patient compliance and outcomes.

